# Comparative study on the effects of sous-vide, microwave cooking, and stewing on functional properties and sensory quality of goose meat

**DOI:** 10.1016/j.psj.2023.103064

**Published:** 2023-08-26

**Authors:** M. Wereńska

**Affiliations:** Department of Food Technology and Nutrition, Wroclaw University of Economics and Business, 53-345 Wroclaw, Poland

**Keywords:** goose meat, heat treatment, functional properties, sensory evaluation, CIE lab

## Abstract

The effect of sous-vide (**SV**), microwave (**M**) cooking, and stewing (**S**) on selected functional properties of goose meat were investigated in this study. It was measured cooking loss (**CL**), texture and color parameters, and sensory evaluation was carried out. The material were 96 breast muscles (**BM**, *n* = 48 with skin and subcutaneous fat and *n* = 48 without skin) from 17-wk-old “Polish oat geese.” The kind of heat treatment and the type of goose meat and interaction the type of meat × heat treatment affected the amount of CL. The lowest value of CL was stated for SV samples. The meat with skin was characterized by a lower shear force value (**SF**), hardness, gumminess, and chewiness than samples without skin for all cooking methods. There were no differences in SF value for M and S samples with skin. The S samples characterized by the highest value of SF, hardness and the SV meat by the lowest for both kind of meat. The M samples characterized by the higher value of cohesiveness, gumminess and chewiness compare to SV and S meat. All instrumental color parameters were significantly affected by cooking technology (*P* = 0.001). There were differences in color lightness (L*) of investigated cooked samples. The SV meat had the highest value of L* parameter and was characterized by a lighter color among others. The highest decrease in a* value was stated for S and lowest for SV meat. The SV showed more intense red color than remaining samples. The a* value decreased and b* parameter increases with an increase the heat treatment temperature. The value of b* was higher in S (about 100°C) samples than in raw meat and processed by SV (70°C). From consumer point of view the best color had goose breast muscles subjected to SV process. Considering all tested sensory features, the overall palatability of SV goose samples was rated as excellent, S as very good and M as good.

## INTRODUCTION

A good source of protein and lipids for humans is, among others, chicken and turkey meat, which are the most popular in all world countries. Also meat of waterfowl, including the goose meat, is a good source of protein and lipids. Goose meat is generally consumed in much smaller amounts than turkey, chicken pork, beef and lamb. Despite its culinary value, specific nutritional due to its high price, it is less popular than gallinaceous poultry meat. It is well known that goose meat provides fat with a high content of MUFA with one of the highest Ʃ UFA/Ʃ SFA ratio among the various types of meat and therefore it can be a valuable and desirable foodstuff for the consumer (Boz et al., 2019; Goluch et al., 2021). To source and sell this meat commercially in the global market, domestic geese (various breeds and types) and wild geese are used (Geldenhuys et al., 2016; Kozák, 2019). In Poland, the most popular breeds are White Koluda geese, often called “Polish oat geese.” The birds are fattened freely with oats in the last 3 wk of rearing and therefore the meat is characterized by a specific, delicate palatability and desirable tenderness (Nowicka et al., 2018). Meat and meat-based products are cooked before being eaten, heat treatment guarantees a safe product. The meat can be prepared in a variety of ways to meet the consumers’ flavor preferences. Generally, consumers choose a cooking method that produces high-quality meat products having a favorable color, texture and taste (Pathare and Roskilly, 2016). Therefore, the method of heat treatment of meat is extremely important. Cooking promotes a cascade of adverse changes in chemical composition and functional properties of meat. In addition, some thermal processes carry a risk of the formation of substances harmful for humans. Therefore, people's preferences have changed to minimally processed, easy-to-eat foods with the least additives, too. The alternative to the most popular heat treatment methods for goose meat may be sous-vide cooking, resulting in a product classified as “ready to eat” (**RTE**). The technique called sous-vide is one of the most popular forms of LT-LT (low temperature 50°C–85°C, long time 2–48 h) cooking in a bag, which applies the principle to vacuum-packed meat with water or steam as heating media (Baldwin, 2012; Zielbauer et al., 2016; Dominguez-Hernandez et al., 2018; Gómez et al., 2020). Sous-vide cooking differs from traditional cooking methods in 2 fundamental ways: the raw food is vacuum-sealed in heat-stable, food-grade plastic pouches, and the food is cooked using precisely controlled heating. Vacuum-sealing has several benefits: it allows heat to be efficiently transferred from the water (or steam) to the food; it increases the food's shelf-life by eliminating the risk of recontamination during storage; it inhibits off-flavors from oxidation and prevents evaporative losses of flavor volatiles and moisture during cooking (Baldwin, 2012). This method improves the juiciness, color retention, and flavor of the meat and minimizes nutrient loss while optimizing its palatability (Díaz et al., 2008; Roldán et al., 2013; Kato et al., 2016; Zielbauer et al., 2016; Falowo et al., 2017; Jeong et al., 2018). Sous-vide has a positive effect on the tenderness of the meat, as well. Tenderness of cooked meat is one of the main quality criteria and usually associated with heat-induced deformation of muscle tissues comprising collagenous and myofibrillar proteins (Christensen et al., 2011). Cooking at low temperatures lowers protein-protein association and gelation and increases the water holding capacity (Tornberg, 2005). It seems that under these sous-vide conditions, there occurs an intense collagen solubilization, which in turn leads to a great formation of gelatine, while the myofibrillar-based toughening is still not very intense (Sánchez del Pulgar et al., 2012; Roldán et al., 2013; Jeong et al., 2018). The tenderization achieved through sous-vide cooking is mainly attributed to the reduced denaturation of proteins at the typically lower temperatures used, weakening of connective tissue through collagen solubilization, and retention of water (Christensen et al., 2013; Jeong et al., 2018; Naqvi et al., 2021). The color measurement in cooked meat can provide reliable information about eating quality attributes. Myoglobin is the main heme pigment responsible for meat color. The other species contributing to color changes during the cooking of meat are deoxymyoglobin (**DeoxyMb**), oxymyoglobin (**OxyMb**), metmyoglobin (**MetMb**), and sulfmyoglobin (**SulfMb**). During cooking, 3 forms of myoglobin are converted and degraded through oxygenation, oxidation, and reduction reactions, ultimately influencing the appearance of meat color. The higher the temperature, the higher the change in myoglobin (García-Segovia et al., 2007; Wołoszyn et al., 2020). The disadvantages of sous-vide cooking are the high cost of equipment, including an immersion circulator and a vacuum sealer, and high energy consumption during prolonged cooking (Karpińska-Tymoszczyk et al., 2020). It should be noted that the information on the application of the sous-vide technique in goose meat is limited and needs to be improved. Given the above, this study aimed to compare the effect of sous-vide, microwave cooking, and stewing on the functional properties such as cooking loss (**CL**) color parameters (L*, a*,b*, C, h), Warner-Bratzler shear force (**SF**), texture profile parameters (hardness, springiness, gumminess, cohesiveness, chewiness), and sensory quality of goose meat. This paper complements our previous studies (Wołoszyn et al., 2020; Goluch et al., 2021; Wereńska et al., 2021, 2023a,b) on the effect of various heat treatment methods on the quality of goose meat. The comparison of these cooking methods is important as it provides information for consumers and industrial practices.

## MATERIALS AND METHODS

### Meat Samples

The material for the study were boneless, goose breast muscles (**BM**) (*Pectoralis major*) from White Kołuda geese (Polish oat geese) which were slaughtered after 17 wk of life and delivered by the producer. The 96 breast muscles (*n* = 48 with skin and subcutaneous fat and *n* = 48 without skin) were investigated. The birds were reared on the same commercial farm and fed on the same complete concentrated diet (Wołoszyn et al., 2020). Before the slaughter, geese were subjected to feed withdrawal for 12 h. Next, the birds were slaughtered in a poultry plant, according to the EU regulations (Council Regulation EC No 1099/2009). The raw material was stored in conditions recommended by the producer (+4°C ± 1°C, 24 h), washed, trimmed, and calibrated to 25 to 27 mm of thickness. One serving portion of the breast with skin was 472 ± 13 g, and without skin 354 ± 35 g. Then the raw material was subjected to thermal treatment and was investigated. Before heat treatment, the color parameters L* (lightness), a* (redness), and b* (yellowness) were measured in raw material.

### Heat Treatments

It was used the same thermal heating conditions as in our previous 2 experiments (Wereńska et al., 2023a,b), as the current study complements them. For this purpose sous-vide, microwave cooking and stewing methods were tested. A total of 32 breast muscles (8 samples with skin and 8 samples without skin were investigated twice) of geese were used in each kind of heat treatment. After the completed thermal processes, the muscles were cooled to room temperature and placed in a refrigerator at 4°C for 24 h. After this time, the muscles were removed from the refrigerator, allowed to reach ambient temperature in an atmosphere of air (21°C, 3 h) and they were tested. The CL, color parameters such as L *, a *, b *, Chroma (C), hue angle (h^o^) and the individual differences (ΔE) in L*, a* and b* values, SF, texture profile analysis (**TPA**—hardness, springiness, cohesiveness, gumminess, chewiness) and sensory evaluation (flavor and aroma typical for goose meat, tenderness, juiciness, cohesiveness, springiness and overall palatability) were determined.

### Sous-Vide

In the study, for the sous-vide (**SV**) cooking method, the temperature of 70°C and time 4 h (240 min) was used. Prior to cooking, each breast muscles was weighed, placed into a vacuum polyamide/polyethylene pouches (thickness of 92 μm, heat resistance (**HR**) of -40°C/+120°C, O_2_ permeability of 9 cm^3^/m^2^ per 24 h at 4°C/80% HR and water steam permeability of 1.2 g/m^2^ per 24 h) and further heat-sealed using vacuum sealing machine (Profi Line 40+, Hendi, Robakowo, Poland) with extent of vacuum 99.6%. After that, the samples were submerged in a thermostated water bath (model SW 22, Julabo GmbH, Seelbach, Germany) that was preheated to 70°C. The heating time of 4 h was applied once the core temperature of the muscles reached the water bath temperature of 70°C (the hand-held thermometer was used in an additional control sample-Thermometer, DT-34, Termoprodukt, Bielawa, Poland). Immediately after the heat treatment, the pouches were removed from the water bath and rapidly chilled with ice-cold water (2°C) for 1 h. Thereafter the packed breast muscles were put in chilled storage at 4°C for 24 h.

### Stewing

Before stewing (**S**), the muscles were first pan-fried with goose lard (10 g) for 1 min per side. Thereafter the samples were placed individually (without bags) on a stainless-steel pan and added 250 mL of hot water (the muscles were covered with water), covered with a lid and kept on the cooker for approximately 1 h 30 min (90 min, core temperature was about 100°C).

### Microwave Cooking

Microwave cooking (**M**) was performed using Whirlpool MWP 253 SX (Whirlpool EMEA, Wroclaw, Poland) microwave oven equipped with a revolving plate. Each breast muscle was separately placed on a ceramic container (without bags) in the center of carousel, and cooked at 700 W, for 8 min (2 heating cycles of 4 min on each side), to reach a final core temperature about 75°C, required to achieve a constant degree of the center of probe. It was checked by placement in the center of each muscle Teflon-coated thermocouple (Type T, Omega Engineering Inc., Stamford, CT) combined with a temperature recorder (VAS Engineering Inc., San Diego, CA).

### Functional Properties

#### Cooking Loss

To calculate the percentage of cooking loss, the bags (when the sample cooled down to room temperature) were opened, and breast samples were weight. CL was calculated from differences in the weights before (W) and after heat treatment (W_h_). It was 3 repetitions from each muscle (Wołoszyn et al., 2020).

$$\text{CL}\left(\%\right)=\left(W-W_{h}\right)/W\times 100\%$$

#### Color Parameters

Color determination of raw and cooked samples was carried out using a Minolta Colorimeter (model CR-310, Konica Minolta Co. Ltd, Osaka, Japan) equipped with a standard illuminant D_65_ and 8 mm viewing port. The measurements are presented in the CIE L*, a*, b* color scale (CIE, 1986). The assessments were carried out on 3 preselected locations at the surface of each sample. Before measurement, the apparatus was calibrated according to the white reference standard Y = 94.2; x = 0.313; y = 0.324. L* (lightness, black = 0, white = 100), a* (redness >0, greenness <0), b* (yellowness, b*>, blue <0), C (Chroma, 0 at the center of the color sphere), and hue° (hue angle, red = 0°, yellow = 90°, green = 180°, blue = 270°) were quantified on each samples using a 10° position of the standard observer. Chroma (C), hue angle (h°), and the individual differences (ΔE) in L*, a* and b* values were calculated using the following equations (CIE, 1986):

$$C={\left(a^{*2}+b^{*2}\right)}^{1/2}$$

h° = tg^−1^ (b*/a*), were h° = 0° for reddish hue and h° = 90° for yellowish hue.

$$\Delta E={\left[{\left(\Delta L^{*}\right)}^{2}+{\left(\Delta a^{*}\right)}^{2}+{\left(\Delta b^{*}\right)}^{2}\right]}^{1/2}$$

#### Warner-Bratzler Shear Force and Texture Profile Analysis

Warner-Bratzler peak SF was measured using a Warner-Bratzler shear force blade (V-shaped) attached to an Instron Universal Testing Machine (model 5543, Instron Corp. Canton, Norwood, MA). Each cooked sample was subdivided into 6 round cores of approximately 2.54 cm diameter × 1.0 cm height, ensuring the orientation of muscle fibers was parallel to the long edge of the block and perpendicular to the blade. The meat cylinders were sheared in the texturometer (was used to test crosshead speed of 50 mm/min) and the mean of the peak shear force from 6 replicates from each experimental samples was used as an estimate of shear force (expressed in N).

TPA was conducted methods published by Bourne (1978) and Bourne (2002) at room temperature with Instron Universal Testing Machine (model 5543, Instron Corp. Canton, Norwood, MA). TPA includes hardness, springiness, cohesiveness, gumminess, chewiness. From each muscle, 3 samples (2.54 cm diameter × 1.0 cm height) parallel to the longitudinal orientation of the muscular fibers were taken. A 5.7 cm diameter flat-ended probe was attached to a texture analyzer and the sample was oriented to ensure fibers were perpendicular to the direction of the probe. The force required and work done to drive a flat-ended probe to compress the sample to 70% of its height was recorded. The instrument was set with a crosshead speed of 5 mm/min and wait time of 5 s in a double-bite compression test. The TPA parameters were calculated using Bluehill 3—testing Software Instron.

### Sensory Evaluation

The sensory quality of heat treatment goose meat was conducted using a quantitative descriptive analysis (**QDA**) (Stone et al., 2020). A panel of 7 judges, based upon previous experience with sensory analysis of poultry meat (trained according to ISO 3972, 2011 procedure) was selected. The team consisted of nonsmokers (all female in age 33–52). The sensory evaluation was carried out at the Sensory Laboratory located in the Department of Food Technology and Nutrition in Wroclaw (Poland) with all requirements the international standards EN ISO 8589:2010 (2010). Samples were cut into cubes (1.5 × 1.5 × 1.5 cm), labeled with assigned 3-digit codes and served randomly to the panelists on the same white glass plates. Unsalted crackers and distilled water were provided to clean and refresh the palate between samples. Selection of quality descriptors for quantitative descriptive analysis was carried out in accordance with the ISO procedure (ISO 13299, 2016).

Each breast muscles was evaluated in terms of flavor and aroma (typical for goose meat), tenderness, juiciness, cohesiveness, springiness and overall palatability. The samples were analyzed for the intensity of sensory descriptors. It was 3 repetitions from each muscle.

To determine intensity of each sensory attribute, a 10-point scale expressed in conventional units (**CU**) with specific word anchors was used. List of definition for quality attributes is presented in Table 1 and the same as in previous our study (Wołoszyn et al., 2020).The opinions expressed by each assessor were recorded on an evaluation sheet that had been created during previous tasting sessions, in which panelists selected and defined the specific attributes of goose meat.

**Table 1:** Descriptors used in sensory evaluation and definitions of points scale (CU).

Score	Flavor and aroma typical for goose meat	Tenderness	Cohesiveness	Juiciness	Springiness (elasticity)	Overall palatability
0–2	Absence (extremely low intensity)	Extremely tough	Extremely low (extremely easy to fragmentation)	Extremely dry	No elasticity	Unacceptable
3–4	Sufficiently	Moderately though	Low	Moderately dry	Low elasticity	Sufficient
5–6	Pleasant-good	Moderately tender	Moderately low	Moderately juicy	Moderately elasticity	Good
7–8	Very pleasant	Tender	High	Juicy	Elasticity	Very good
9–10	Excellent (extremely high intensity)	extremely tender	extremely high (extremely difficult to fragmentation)	Very juicy	Extremely elasticity	Excellent (extremely desirable)

### Statistical Analysis

The data were analyzed as a completely randomized design using a 2-way ANOVA concerning the kind of muscles (with and without skin) and heat treatment (stewing, microwave cooking, and sous-vide) as a factorial design (2 × 4), according to the following linear model: Y_ij_ = μ + A_i_ + B_j_ + (AB)_ij_ + e_ij_, where Y_ij_ = value of trait (the dependent variable); μ = overall mean; A_j_ = effect of kind of muscle; B_j_ = effect of heat treatment; (AB) = interaction and e_ij_ = random observation error, using Statistica13.3 software (Statsoft Inc., 2019). The statistical significance of the differences between the averages of the groups was calculated using Tukey's test and was at a level of *P* ≤ 0.05. The tables present the average values and their standard deviations. The different letters in rows means statistically significant differences between group average of one parameters, including thermal treatment (*P* ≤ 0.05); and the different letter in columns means statistically significant differences between group average of one parameters, including kind of meat (*P* ≤ 0.05);

## RESULTS AND DISCUSSION

### Cooking Loss

The cooking loss can vary depending on several factors, including the type and cut of meat, cooking method, cooking temperature, and cooking duration. Cooking loss is a combination of liquid and soluble matter lost during cooking. With increasing temperature, the water content decreases while fat and protein contents increase, indicating that the central part of the cooking loss is water (Brugiapaglia et al., 2012; Domínguez et al., 2014), probably due to heat-induced protein denaturation during meat cooking, which causes less water to be entrapped within the protein structures (Juárez et al., 2010). The cooking loss depends on mass transfer during thermal treatment and, therefore, different cooking methodologies will lead to different losses. Moisture losses during dry cooking are due to losses by evaporation, and during wet cooking there are also losses by exudation and diffusion. Cooking methods that involve higher heat and longer cooking times, such as grilling or roasting, may result in higher cooking loss compared to methods that involve lower heat and shorter cooking times, such as steaming or braising (Cheng and Sun, 2008; Oz et al., 2017).

In the present work cooking loss from goose meat were significantly (*P* = 0.001) affected by the cooking method and was in the range 22 to 43% for meat without skin and 32 to 52% for meat with skin. In experiment both, the kind of heat treatment and the type of goose meat (with and without skin and subcutaneous fat) affected (*P* = 0.001) the amount of cooking loss (Table 2), there was also interaction between the type of meat and heat treatment (*P* = 0.006). The breast goose meat with skin and subcutaneous fat characterized higher CL than that without skin, because, an additional component loss during heat treatment for goose breast muscles with skin is subcutaneous fat. It was similar to our previous results (Wołoszyn et al., 2020) obtained for boiled, oven-convection roasted, pan fried and grilled goose meat. The S goose meat was characterized by higher CL than M and SV meat (*P* = 0.001) for both kind of meat.

**Table 2:** Cooking loss, shear force, and TPA parameters of goose meat (*n* = 16 breast muscles with skin and *n* = 16 without skin for each kind of heat treatment).

Parameters	Meat	Sous-vide (SV)	Microwave (M)	Stewing (S)	Total	*P* values (*P* ≤ 0.05)		
						Meat (Mt)	Heat treatment (T)	Mt × T
Cooking loss (%)	Without skin With skin Total	y22.11^c^ ± 1.06	y34.19^b^ ± 1.89	y43.07^a^ ± 2.06	y33.13 ± 8.99	0.001	0.001	0.006
		x32.21^c^ ± 2.81	x46.13^b^ ± 3.75	x52.19^a^ ± 2.06	x43.51 ± 9.03			
		27.16^c^ ± 5.65	40.16^b^ ± 6.85	47.63^a^ ± 5.13				
Shear force (N)	Without skin With skin Total	x62.41^c^ ± 6.04	x71.77^b^ ± 5.09	x82.80^a^ ± 6.73	x72.33 ± 10.26	0.001	0.001	0.055
		y51.93^b^ ± 3.66	y63.87^a^ ± 4.99	y64.84^a^ ± 3.60	y60.21 ± 7.18			
		57.17^b^ ± 7.25	67.82^a^ ± 5.54	73.82^a^ ± 11.39				
Hardness (N)	Without skin With skin Total	x249.73^b^ ± 6.20	x276.57^a^ ± 7.93	x284.08^a^ ± 8.06	x270.12 ± 16.71	0.001	0.001	0.100
		y229.42^b^ ± 7.56	y240.59^a^ ± 10.02	y252.67^a^ ± 5.32	y240.89 ± 12.25			
		239.57^c^ ± 9.50	258.58^b^ ± 20.67	268.38^a^ ± 17.65				
Cohesiveness (ratio)	Without skin With skin Total	0.50^c^ ± 0.01	0.57^a^ ± 0.02	0.53^b^ ± 0.01	0.52 ± 0.05	0.016	0.001	0.007
		0.51^b^ ± 0.01	0.56^a^ ± 0.02	0.52^b^ ± 0.01	0.51 ± 0.07			
		0.50^b^ ± 0.01	0.56^a^ ± 0.02	0.52^b^ ± 0.03				
Springiness (ratio)	Without skin With skin Total	0.56 ± 0.03	0.61 ± 0.07	0.59 ± 0.05	0.58 ± 0.06	0.379	0.039	0.869
		0.55 ± 0.04	0.60 ± 0.05	0.56 ± 0.03	0.57 ± 0.04			
		0.56^b^ ± 0.03	0.61^a^ ± 0.06	0.58^a^^b^ ± 0.04				
Gumminess (N)	Without skin With skin Total	x123.63^c^ ± 3.95	x156.66^a^ ± 5.02	x137.34^b^ ± 5.56	x139.21 ± 14.68	0.001	0.001	0.009
		y115.86^c^ ± 4.10	y134.71^a^ ± 7.61	y130.15^b^ ± 4.66	y126.91 ± 9.83			
		119.75^c^ ± 5.58	145.69^a^ ± 13.01	133.74^b^ ± 6.17				
Chewiness (N)	Without skin With skin Total	x68.59^c^ ± 4.34	x97.55^a^ ± 11.33	x78.41^b^ ± 7.17	x81.52 ± 14.54	0.001	0.001	0.603
		y63.59^c^ ± 4.75	y84.34^a^ ± 5.96	y69.48^b^ ± 1.96	y72.47 ± 9.94			
		66.09^c^ ± 5.06	90.94^a^ ± 11.06	73.95^b^ ± 6.84				

a–c Different superscript letters in rows mean statistically significant differences between group average, including thermal treatment (*P* ≤ 0.05);

x–y Different superscript letters in columns mean statistically significant differences between group average, including kind of meat (*P* ≤ 0.05). ± Standard deviation.

The lowest value of CL was stated for SV samples. Cooking loss is known to proportionally increase with increasing temperature and to depend on the final temperature at the center, too.

Increased temperatures during cooking cause myofibrillar proteins to shrink, a process that starts at 40°C and becomes more intense with increased temperatures. Due to this shrinking process, a parallel decrease occurs in the interfibrillar volume, which in turn reduces the myofibril's ability to hold water. Consequently, part of the water retained by capillarity is lost during cooking. The increase in cooking loss along with the increase in temperature is attributed to collagen and actin denaturation. At heat process, to take place contraction of the perimysial connective tissue causing a compression of the muscle fiber bundles, which in turn encourages water to be released from the meat cut (Sánchez del Pulgar et al., 2012; Modzelewska-Kapituła et al., 2019). In present study, the final internal temperatures reached were significantly different among the cooking processes and the cooking losses were also affected by the cooking method used. In case S, the heating temperature, internal core temperature were higher than in case M and SV samples. Unlike the other cooking methods, the lower temperature condition in SV minimizes the coagulation of heat-sensitive proteins (Dominguez-Hernandez et al., 2018). The second cause for lower cooking losses for SV samples was heating the meat in a sealed plastic pouch. The package prevented water removal from meat samples as it served as a physical barrier and it held moisture inside the bag, preventing also the discharge of liquid due to limited space (Baldwin, 2012; Rinaldi et al., 2014). Whereas microwave cooking is the main energy source, to render the fat and coagulate the proteins by increased temperature. In the same time the surface water is removed by a connective air flow. These results corroborate with the previous obtained by Oz and Celik (2015) for CL from goose breast muscles subjected to microwave cooking. They observed higher value of CL for microwave cooked meat compared to grilled, but lower than boiled, fried with oil and deep fat-fried samples. In the study of Domínguez et al. (2014) with cooked foal meat, the kind of heat treatment significantly affected CL. The cooking loss was significantly higher (32.5%) after microwaving and lower after grilling (22.5%) and frying (23.7%). Alfaia et al. (2010) observed for beef meat, higher CL during microwaving than boiling and grilling. They suggested that the absence of crust formation caused the higher CL in microwaved meat. In the study of Nikmaram and Emam-Djomeh (2011) and Nikmaram et al. (2011) the overall percentage loss due to cooking for veal and camel meat cooked by microwave was more (about 42 and 43%, respectively) than conventional methods (about 32 and 30% for braising, about 34 and 32% for roasting, respectively). They concluded it was likely that the high electromagnetic field, and short time to final temperature associated with microwaving cause rapid protein destruction and disintegration of the texture matrix, which resulted liberalization of large amounts of water and fat. However, braising and roasting require a long time to reach the final temperature, which does not shock proteins and limits the amount of cook loss. Braising increases the opportunities for dissolving intracellular materials into the cooking liquid, whereas roasting gradually creates an external hard layer around the sample that slows the seeping liquid's exit. In contrast, Choi et al. (2016) indicated that the CL of chicken steaks was lower for microwaving than the connective oven roasting, grilling, and boiling. According to Maranesi et al. (2005), microwave cooking of the lamb rib-loins produced the lower CL (27.1%) than broiling (36.5%) and it is in accordance with results presents for goose meat. The results of Abdel-Naeem et al. (2021) indicated that rabbit meat cooked by boiling and pan-frying exhibited the most significant CL, whereas, grilling- and microwave cooked samples induced the significant (*P* < 0.05) lower loss. According to Macharáčková et al. (2021), the CL from pork muscle in a range 9.7 to 32.5% was noted after SV cooking (different temperature and time was used) and 28.1% after broiling (200°C/5 min). The differences were stated between the individual temperatures used for the SV and the losses found. The same CL were found after the treatment by broiling and after SV at a temperature of 70°C/2 h. The lowest cooking losses were noted for SV at 55°C, which differ from all the other types of SV as well from broiling. Sánchez del Pulgar et al. (2012) observed that cheek pork samples cooked in boiling water in a sauce pan in the traditional manner also showed more CL than vacuum packaged meat cooked at 60°C, and yet, the samples in the traditional method had similar value to that obtained after cooking in plastic bags at 80°C. On the other hand Domínguez et al. (2014) and Lorenzo et al. (2015) using roasting, grilling, microwaving baking and frying with olive oil for foal meat stated that the CL was as follows: microwaved > roasted > fried > grilled. In the study of Haghighi et al. (2021), chicken breast muscles cooked SV method (at different temperatures) and in water bath (100°C/60 min), were characterized of CL in ranged 10.23% (SV 60°C/60 min) to 28.08%. Cooking loss was affected by both cooking temperature and cooking time and it was increased by increasing cooking temperature and time (*P* < 0.05). Modzelewska-Kapituła et al. (2019) stated that SV treatment of beef meat caused lower (*P* < 0.001) CL than steam cooking (30.0 vs. 34.2%, respectively). Soletska and Krasota (2015) observed that chicken fillets which were made by SV technology (65°C/60 min) decreased cooking loss in 4 times compare to the traditional boiling in water at 98°C/30 min. Likewise Głuchowski et al. (2020) showed that salmon processed with SV method was characterized by a higher yield (90.9–93.7%) than the roasted and steamed (83.8 and 88.4%) methods. In an experiment by Park et al. (2020) was, marked differences (*P* < 0.05) in cooking loss between the breast muscles cooked in a convection oven (control sample) at 180°C temperature and SV groups (6 combinations of cooking temperature and time were used). The control sample showed a higher CL (26.5%, *P* < 0.05) than all sous-vide treatments (6.6–25.3%). Also, Przybylski et al. (2021) observed that chicken meat after SV treatment was characterized by a significantly lower CL (by 17.5%) than that for water bath cooking. Głuchowski et al. (2020) stated that, the yield of chicken breasts processed with the SV method at different temperatures (64°C, 66°C, 75°C) was significantly higher (82.4–89.4%; *P* ≤ 0.05) than after heat treatment with traditional (steaming, boiling) methods (72.4 and 69.5%, respectively), where the temperature was higher. In the study of Polak and Markowska (2019), the highest CL was stated in water bath cooked turkey meat (23%), while in the case of the SV technology, it was significantly lower and amounted to 3 to 16% depending on the used temperature and the time of thermal treatment. As shown above, the different cooking methods and conditions, the material used in experiments, and various other factors, which often need to be reported, caused the results concerning the cooking loss to vary significantly. Taking into account the efficiency of the heat treatment process and consumer acceptance, the sous-vide method seems to be the best among the tested methods.

### Color Parameters

During heating, the changes associated with sensory attributes of food products can reflect texture and color properties. Food color changes during thermal processing can be influenced by degradation of pigments, oxidation of ascorbic acid, enzymatic browning, and nonenzymatic browning (Ling et al., 2015; Guo et al., 2017). It is well known that the denaturation of proteins coming during heat treatment of meat affects color changes. An increased reflection of light arising from light scattering by denatured proteins causes lesser lightness, while thermal denaturation of myoglobin and other proteins causes the least redness. The color difference between the meat cooking methods is due to changes in the myoglobin pigment. During cooking in range temperature of 55°C and 65°C, denaturation of the protein portion in myoglobin, and next, most of the myoglobin is denatured and brown at 70°C to 80°C. However, sensitivity to heat denaturation differs among the 3 forms of myoglobin. While the 3 forms of myoglobin differ in their sensitivity to heat denaturation, deoxymyoglobin, which is dominant in vacuum-packaged meat, is more resistant to heat denaturation than oxymyoglobin and metmyoglobin (Van Laack et al., 1996; Hunt et al., 1999; King and Whyte, 2006; Park et al., 2020).

Table 3 displays the obtained instrumental color parameters of lightness (L*), redness (a*), and yellowness (b*), as well as the calculated hue angle (h^o^) and Chroma (C) for goose meat cooked under the different experimental conditions studied. All instrumental color parameters were significantly affected by cooking technology (*P* = 0.001). The analysis of meat color revealed differences in color lightness L* (40.28–44.44) of all investigated cooked samples, but generally, only minor differences in the L* values between the different heat treatments were observed. The meat samples become lighter after heat treatment was due to the increase reflectance and scattering of light by denatured proteins and aggregated sarcoplasmic and myofibrillar proteins (Young and West, 2001; Christensen et al., 2011). In our study a significant decrease (22.13 vs. 8.92) in redness (a*) was noted for all cooked goose meat compare to raw sample, but for b* parameter it was stated significant higher value (11.28 vs. 2.52). According to King and Whyte (2006) the value of parameter a* in cooked meat is inversely proportional to the degree of myoglobin denaturation. The highest decrease in a* value was stated for S (by 59.7%—the heating temperature was 100°C) and lowest for SV meat (the heating temperature was 70°C). The SV showed a more intense red color than the remaining samples. Generally, in the present study, the a* value decreased with increased the heat treatment temperature. This observation could be attributed to more advanced myoglobin degradation in S meat. There was an increase of b* parameter as a consequence of cooking temperature. In this experiment, the temperature showed significant effects on a* and b* values, indicating a trend toward lower a* and higher b* values at surface of geese’ breast muscles with higher heating temperature. The value of yellowness b* on the surface of cooked meat was significantly higher in S samples than in raw meat processed by SV and M technology. Thermal processing denatures myoglobin and leads to the formation of metmyoglobin in meat. Metmyoglobin undergoes further denaturation, resulting in a brown color. Heat generally causes the denaturation and unfolding of the globin molecule, resulting in globin-hemichrome or ferrihemochrome which is known as the dull-brown pigment formed during heating (Zhang and Wang, 2012; Roldán et al., 2013; Suman and Joseph, 2013). The higher cooking temperature probably induced further denaturation of metmyoglobin. In other studies presented by García-Segovia et al. (2007), Christensen et al. (2011), Roldán et al. (2013), and Becker et al. (2016), the contribution of yellowness (b*) in beef, pork, and lamb color increased with an increase in cooking temperature. The results for goose meat are in line with a* and b* values with previous findings obtained for cooked pork meat by Christensen et al. (2011) Sánchez del Pulgar et al. (2012) and Becker et al. (2016).

**Table 3:** CIE Lab—color parameters of goose meat (*n* = 96 breast muscles—raw meat, *n* = 32—cooked breast muscles for each kind of heat treatment).

Parameters	Raw meat (R)	Heat treatment			Total	Heat treatment (*P* ≤ 0.05)
		Sous-vide (SV)	Microwave (M)	Stewing (S)		
L*	39.59^b^ ± 3.88	44.44^a^ ± 3.43	43.45^a^ ± 3.42	40.28^b^ ± 4.06	42.13 ± 4.13	0.001
a*	22.13^a^ ± 2.11	12.34^b^ ± 1.05	9.94^c^ ± 1.28	8.92^d^ ± 1.08	12.62 ± 5.06	0.001
b*	2.52^c^ ± 0.35	9.88^b^ ± 0.77	9.24^b^ ± 0.85	11.28^a^ ± 1.07	8.57 ± 3.21	0.001
C	22.28^a^ ± 2.10	15.83^b^ ± 1.05	13.62^c^ ± 0.96	14.41^c^ ± 1.25	16.01 ± 3.48	0.001
h°	6.53^d^ ± 0.97	38.71^c^ ± 2.84	43.04^b^ ± 4.94	51.67^a^ ± 3.52	37.04 ± 16.23	0.001

a–d Different superscript letters in rows mean statistically significant differences between group average, including thermal treatment (*P* ≤ 0.05). ± Standard deviation.

The cooking changed the hue angle (h^o^), and Chroma (C) parameters of goose meat, too (Table 3). Chroma or meat color saturation relates to the concentration of myoglobin and its degree of denaturation. This attribute is more predominant with higher concentrations of myoglobin and at a lower rate of denatured myoglobin (Ledward, 1992). Heat treatment caused the reduction in C parameter compared to raw meat (22.28 vs. 13.62). The lower C value for cooked samples showed that they were brighter (less distant to the L* axis of the CIE Lab system) than raw ones. In all cooking samples value of h° parameter was significant higher (38.71–51.67) than raw meat (6.53) and this means that the color of these samples was more yellow (closer to the b* axis of the CIE color space). In cooked meat, the highest L*, C and lowest h° values were stated for SV samples. Hue angle (h°) is affected by the chemical state of the myoglobin and is inversely related to the a* value. Thus, samples SV showed the lowest h* values, most likely due to the lesser degree of myoglobin denaturation mentioned above. Sous-vide meat showed slightly higher L* value than M, but the difference was not significant. The lowest L*, and highest h° for S was observed, however differences in C value for M and S goose breast muscles were not significant. Sánchez del Pulgar et al. (2012) stated that the Chroma values for pork samples SV 60°C were higher than those obtained for samples cooked at 80°C and that cooked in boiling water. The samples SV 60°C showed lower h* values than those cooked at 80°C and those cooked in boiling water, most likely due to the lesser degree of myoglobin denaturation.

There is in the literature a controversial discussion concerning L* value changes during increasing temperature heating. Some authors describe lower L* values (Sánchez del Pulgar et al., 2012; Roldán et al., 2013), while others describe higher L* values at higher temperatures (García-Segovia et al., 2007; Christensen et al., 2011). The presented results of L* value for goose meat are accordance with data detected (for pork and lamb meat) by Sánchez del Pulgar et al. (2012) and Roldán et al. (2013). Sánchez del Pulgar et al. (2012) deduced that all the color parameters were affected by cooking temperature, too. Thus, pork meat SV 60°C showed higher L* values than those SV 80°C and those cooked unpackaged by means of the traditional method in boiling water. This result may be related to the higher amount of exuded water, which remains impregnating the surface of the water. This occurrence has been pointed out as a cause of higher L* values. Roldán et al. (2013) described that the lamb samples cooked at SV 60°C showed slightly higher L* values than those cooked at 70°C or 80°C. Christensen et al. (2011) observed that L* values increased with increasing temperatures while increasing time did not affect L* values in *Longissimus dorsi* (**LD**) from slaughtered pigs and sows. In LD from sows, a* values decreased with increasing temperature. In a study by García-Segovia et al. (2007), the color analysis suggested that the cooked beef steaks were generally lighter (higher L*) and more yellow (higher b*), whereas a* (red color) decreased as temperature and cooking time increase. They stated that meat cooked at the cook-vide (**CV**) system had L* value significantly higher than the samples cooked at atmospheric (A) or SV conditions. In contrast the a* and b* values were higher for SV meat than A and CV meat. The values calculated for C (Chroma) were between 6 for a raw sample and 16 in SV treatment, h° oscillated between 59 for raw and 83 for SV samples. In addition, the higher h° values for meat indicated that the color was more yellow than the remaining samples. In comparison, the higher C values of the SV samples indicated that they were less bright than CV and A ones. In other studies no influence of kind of heat treatment and temperature on L* value have been observed and so for example Oz and Celik (2015) showed that the cooking method had only significant effect on a* and b* value for goose breast muscles. They observed no significant differences in L* parameter for microwaved, pan fried with oil, boiled, oven roasted, deep fried samples. The cooking process caused a reduction in L* and a* values, while it increased b* value compare to raw meat. In our study the L* and b* values for cooked samples increased and a* decreased compared to raw meat. In turn, Nikmaram and Emam-Djomeh (2011) stated only the differences in color parameters (L*, a*, b*) between raw and heating treatments of veal meat. There was no significant difference between microwaved, braised and roasted veal meat for L*, a*, b*, C*, h* and ΔE parameters. In study of Ismail et al. (2019) L* parameter did not significantly differ among SV 65°C, SV 45°C to 65°C, and water bath cooked beef samples (CON 75°C). Park et al. (2020) showed that, vacuum package chicken meat cooked by SV technology at 60°C showed a redder cut surface than meat cooked by conventional oven (control at 180°C) and vacuum-packaged meat cooked at 70°C although no difference in L* between the control and SV 60 groups. Przybylski et al. (2021) stated that in their experiment with chicken breast meat were no significant differences in L* value between SV 76°C and water bath cooking (**WBC**). The SV meat was redder (higher a* value) and less yellow (lower b* value) than the WBC samples.

In Table 4 there were presented the values of the ΔE parameter. It may be deduced that the color of the cooked samples changed a lot in comparison to raw meat. The value of ΔE for raw and heat treatment samples ranged between 13.17 (SV) and 15.87 (S). The biggest differences in color compared to fresh meat were observed for S and the smallest for the SV method. It means that S color samples changed more and SV less than M compared to the raw samples. The calculated values of the ΔE parameter showing the differences in color between the tested cooking methods were as follows: S&SV > S&M > SV&M.

**Table 4:** Color differences (∆E) in goose meat.

Parameters	Raw meat (R)	Heat treatment		
		Sous-vide (SV)	Microwave (M)	Stewing (S)
Raw meat (R)	x	13.17	14.44	15.87
Sous-vide (SV)	13.17	x	2.67	5.56
Microwave (M)	14.44	2.67	x	3.91
Stewing (S)	15.87	5.56	3.91	x

The lowest difference of the ΔE parameter (2.67) was calculated in SV&M pair samples and according to (Třešňák, 1999) indicated minute or perceptible color differences. The pair S&M (3.91) characterized by not yet discordant or medium color differences. The higher differences of the ΔE parameter (5.56) was established between S and SV samples and it showed medium color differences. According to the CIE Lab classification, no parameter ΔE has reached a value lower than 2 (unnoticeable color) and it means that all pairs had visible differences. In an experiment of García-Segovia et al. (2007) ΔE was between 13 obtained for atmospheric pressure and CV (cook-vide) beef meat and 23 for SV samples. They concluded that observed the variation range in Chroma, hue and ΔE at different temperatures, times and treatments there were not very important. In this experiment were moderate differences in the color of SV, M, and S goose meat.

From consumer point of view the best color had goose breast muscles subjected to the sous-vide process. In case sous-vide products, they are very convenient and classified as "ready to eat" products. The disadvantages for the producer are the high costs of production (to produce consume a lot of energy) and for the consumer high market price.

### Warner-Bratzler Shear Force and Texture Profile Analysis

The values for the different textural parameters included the TPA analysis are shown in Table 2. Both, the kinds of goose meat (with and without skin and subcutaneous fat) and cooking method affected SF value (*P* = 0.001). In case SF parameter, there was no interaction between kind of meat and cooking method (*P* = 0.055). It was observed significant differences in TPA parameters such as hardness, gumminess, chewiness (*P* = 0.001, *P* = 0.001), cohesiveness (*P* = 0.016, *P* = 0.001) for kinds of meat and cooking methods. There was no significant difference in springiness value for the kind of meat (*P* = 0.379), but the cooking methods affected springiness (*P* = 0.039). The analyzed variables such as cohesiveness and gumminess were affected by the interaction kind of meat × heat treatment (*P* = 0.007, *P* = 0.009). The examined goose meat with skin and subcutaneous fat was characterized by a lower SF value, hardness, gumminess, and chewiness than samples without skin for all cooking methods.

There were no differences in the SF parameter for M and S samples for meat with skin and in hardness for both kinds of meat. The S samples were characterized by the highest value of SF, hardness, and the SV meat by the lowest for both kinds of meat. The M samples were characterized by a higher value of cohesiveness, gumminess, and chewiness compared to SV and S meat. Mechanical properties of cooked meat are functions of the myofibrillar mechanical properties and connective tissue network. As heat solubilizes the connective tissue leading to meat tenderization, while denaturation of myofibrillar proteins leads to meat toughening. The whole meat toughness was found to decrease between 50°C and 60°C, which was attributed to changes of the perimysial (mainly collagen) connective tissue, while for myofibrillar proteins (mainly myosin) denaturation occurs at 40°C to 60°C. With subsequent gelation of collagen fibers followed by denaturation of actin at 70°C to 80°C. At temperatures above 60°C up to 80°C toughness increased due to increasing breaking strength of the muscle fibers. Generally, both the cooking temperature and time affect meat tenderness, and the increased cooking temperature has a greater effect on fiber shrinkage than increased cooking time. In this way, toughness development during heating of meat can be explained by structural changes in the meat components (Christensen et al., 2000; Nikmaram and Emam-Djomeh, 2011; Baldwin, 2012; Purslow et al., 2016; Dominguez-Hernandez et al., 2018). The decrease in SF as a result of sous-vide treatment was caused by weakening the structure of connective tissue, and gelatinization of collagen. In sous-vide, heating to 70°C could have created a saturated steam inner the bag and a slight overpressure. This saturated steam atmosphere (improved heat transmission) would cause a greater degree of unfolding and denaturation of sarcoplasmic proteins and myosin, as well as a greater collagen solubilization due to the greater effectiveness for breaking the thermolabile bounds of the fibrous structure(Tornberg, 2005). This can explain the high SF value and hardness of the S heated samples and the significantly lower values of the SV variant. In case of M meat, it is likely that the high electromagnetic field, high power, and short time to final temperature associated with microwaving cause protein denaturation and increase in shear force and hardness. Additionally, during microwave heating, the temperature of meat muscle rises more rapidly as compared to SV and S methods due to internal heat generation. Also the faster cooking cycle (for M), the sharper increase in the temperature gradient between the center and surface temperature might have led to a different thermal denaturation response of the myofibrillar proteins that should play a major role in the observed SF value and TPA parameters of goose meat. Thus, the shorter time and higher temperature of microwave cooking and stewing as compared to sous-vide in the present study might have resulted in a lower degree of collagen denaturation and a higher extent of denaturation of myofibrillar proteins, causing higher SF and hardness values. Póltorak et al. (2015) and Jouquand et al. (2015) showed that the SF of bovine meat cooked in a microwave oven was significantly higher than those roasted with traditional convection heating. While, Choi et al. (2016) reported the hardness, gumminess, chewiness of chicken steak cooked in a microwave oven were lower than those cooked by boiling and grilling. Springiness and cohesiveness were not significantly different among all treatments (oven roasting, microwave cooking, grilling, boiling, superheated steaming). In LD of veal cooked by microwaving, roasting, and braising, SF increased and tenderness decreased, but there were no significant differences (Nikmaram and Emam-Djomeh, 2011). The conventionally fried chicken breast muscle had a higher hardness value than the microwaved sample (Mazı and Mazı, 2017). The boiling and pan-frying of rabbit meat provoked the most significant higher shear force values (36.9N and 36.5N), on the contrary, grilling (31.5N), and microwave (31.6N) cooking induced the lowest values (Abdel-Naeem et al., 2021). The highest value of SF was reported by Jeong et al. (2018) for the control pork ham sample boiled in a pot for 45 min than for SV 61°C and SV 71°C. García-Segovia et al. (2007) stated the highest hardness for CV and atmospheric pressure samples (A) compared to SV meat. They considered that this difference could be explained by the greater collagen solubilization and the gel formation with a smaller degree aggregation, in sous-vide. In report of Silva et al. (2016) no significant differences in hardness parameters were noted between grilled and fried chicken charqui samples. Whereas SV promoted a marked decrease in hardness. This hardening decrease they explain the low water losses of sous-vide and a greater collagen solubilization due the long cooking time applied during sous-vide technique. According to Rinaldi et al. (2014) SF for SV 75°C beef muscles was significantly lower compared to both boiling (B) and SV 100°C, probably due to its lower end point temperature as well as its higher water content. Cohesiveness and springiness did not significantly differ, while chewiness was significantly higher for B and SV 100°C in accordance with hardness. Roldán et al. (2013) reported significantly lower hardness values for loin lamb SV samples cooked at 60°C, 70°C, and 80°C for 24 h compared to 8 h and 16 h due to an extensive disintegration of the perimysium around muscle bundle. Springiness, cohesiveness, and chewiness were significantly affected by temperature, cooking time, and by the interaction between temperature and time. On the other hand, hardness, gumminess, and SF values were affected by cooking time and by the interaction between temperature and time. Adhesiveness was not affected by any of the studied factors. Park et al. (2020) noted that the SF and hardness values were higher in cooked chicken breast samples from the control group (CG—were cooked in a convection oven set at 180°C to an internal temperature of 71°C) than that SV 60°C—1 h and 2 h treatments. On the contrary, the adhesiveness value was higher for the SV 60°C—1 h than for the CG, although no difference was observed in chewiness between the CG and SV 60°C—1 h. There were no differences in cohesiveness, springiness, and gumminess between all treatments. Modzelewska-Kapituła et al. (2019) reported that beef meat subjected to SV showed lower SF value than those subjected to steam cooking.

As shown above, the results obtained for many authors in different experiments varied significantly. With all processes coming in connective tissue and myofibrillar proteins of meat during cooking, it results very difficult to elucidate the causes behind the detected changes in texture parameters as a consequence of the used methods and temperature of thermal treatment. The processes of transformation of connective tissue and denaturation of myofibrillar protein in the heat treatment of meat samples are very complicated and depend on many factors (among others: methods of cooking, times, temperature of heat treatment, end-point temperature, kinds of meat, and muscle). The degree of protein denaturation, which implies the change in their structure, is mainly related to the rate of heat flux penetration to the surface of the meat portion, as well as the heat conduction inside the product during thermal treatment. This thermal conductivity inside is very different depend on the type of meat and its size, too (Vittadini et al., 2005; Drummond and Sun, 2006; Panea et al., 2008; Yancey et al., 2011; Wołoszyn et al., 2020).

Considering consumer satisfaction and beneficial rheological properties of cooked goose meat, the most appropriate type of heat treatment seems to be sous-vide cooking.

### Sensory Evaluation

The sensory evaluation was presented in Table 5. The kind of goose meat (with and without skin and subcutaneous fat) effected descriptors such as flavor and aroma typical for goose meat, tenderness, juiciness, and overall palatability (*P* = 0.001). The kind of goose meat did not affect sensory cohesiveness (*P* = 0.793) and springiness (*P* = 0.152). The method of cooking had an influence on all sensory descriptors. There was no interaction kind of meat × heat treatment for all parameters. The higher score for tenderness and higher intensity of flavor and aroma typical for goose meat was established for meat with skin compare to skinless ones. Whereas the higher score for juiciness was noted for meat without skin. Despite this fact it was no significant differences in overall palatability for both kinds of meat.

**Table 5:** Sensory evaluation of goose meat (CU) (*n* = 16 breast muscles with skin and *n* = 16 without skin for each kind of heat treatment).

Parameters	Meat	SV	M	S	Total	*P* values (*P* ≤ 0.05)		
						Meat (Mt)	Heat treatment (T)	Mt × T
Flavor and aroma typical for goose meat	Without skin With skin Total	y8.78^a^ ± 0.44	y6.89^c^ ± 0.78	y7.67^b^ ± 0.87	y7.78 ± 1.05	0.001	0.001	0.941
		x9.78^a^ ± 0.44	x7.56^c^ ± 0.53	x8.56^b^ ± 0.73	x8.63 ± 1.08			
		9.28^a^ ± 0.67	7.22^c^ ± 0.73	8.11^b^ ± 0.90				
Tenderness	Without skin With skin Total	y9.11^a^ ± 0.33	y5.11^c^ ± 1.05	y8.11^b^ ± 1.17	y7.44 ± 1.95	0.001	0.001	0.541
		x10.00^a^ ± 0.00	x6.11^c^ ± 0.78	x9.11^b^ ± 0.60	x8.41 ± 1.78			
		9.56^a^ ± 0.51	5.61^c^ ± 1.04	8.61^b^ ± 1.04				
Juiciness	Without skin With skin Total	y8.89^a^ ± 0.60	y5.33^c^ ± 0.87	y6.89^b^ ± 0.60	7.04 ± 1.63	0.001	0.001	0.735
		x9.89^a^ ± 0.33	x6.22^c^ ± 0.44	x7.78^b^ ± 0.67	7.96 ± 1.61			
		9.39^a^ ± 0.70	5.78^c^ ± 0.81	7.33^b^ ± 0.77				
Cohesiveness	Without skin With skin Total	9.67^a^ ± 0.50	7.89^b^ ± 1.45	6.44^c^ ± 0.73	8.00 ± 1.64	0.793	0.001	0.630
		9.78^a^ ± 0.44	8.22^b^ ± 1.30	6.22^c^ ± 0.44	8.07 ± 1.69			
		9.72^a^ ± 0.46	8.06^b^ ± 1.35	6.33^c^ ± 0.59				
Springiness (elasticity)	Without skin With skin Total	9.67^a^ ± 0.50	7.78^b^ ± 0.83	6.22^c^ ± 0.44	7.89 ± 1.55	0.152	0.001	0.842
		9.89^a^ ± 0.33	8.22^b^ ± 0.83	6.44^c^ ± 1.01	8.19 ± 1.62			
		9.78^a^ ± 0.43	8.00^b^ ± 0.84	6.33^c^ ± 0.77				
Overall palatability	Without skin With skin Total	9.44^a^ ± 0.53	6.44^c^ ± 1.01	7.56^b^ ± 0.53	7.82 ± 1.44	0.001	0.001	0.758
		9.78^a^ ± 0.44	7.11^c^ ± 0.60	8.22^b^ ± 0.67	8.44 ± 1.31			
		9.72^a^ ± 0.46	6.78^c^ ± 0.88	7.89^b^ ± 0.68				

a–c Different superscript letters in rows mean statistically significant differences between group average, including thermal treatment (*P* ≤ 0.05).

x–y Different superscript letters in columns mean statistically significant differences between group average, including kind of meat (*P* ≤ 0.05). ± Standard deviation.

The highest score for all sensory descriptors was established for SV (with skin and skinless). As described in Table 1, the panelists evaluated the flavor and aroma (typical for goose meat) of SV samples as extremely high intensive, the texture was extremely tender, the meat was very juicy and extremely elastic. In case of S and M samples, these parameters received significantly lower sensory scores and therefore, the sensory team evaluated SV samples’ overall palatability as excellent, S meat as very good, and M as good. The higher tenderness of SV goose meat as compared with S and M samples corresponded with lower SF and hardness values, whereas higher juiciness corresponded with lower cooking loss in comparison to S and M samples. Admittedly, since most of the volatile aromatic compounds, which affect cooked meat palatability are created at temperatures above 70°C, it thought then SV goose meat flavor and aroma might result from a combination of fatty acid degradation products and nonvolatile compounds (Dominguez-Hernandez et al., 2018). While the extremely high intensity of flavor and aroma typical for goose meat might be caused for SV samples using vacuum pouches, which prevented the losses of these compounds. Additionally, the longer time of SV compared to M and S heat treatment might result in a higher concentration of the compounds and, as a consequence, a higher intensity of flavor and aroma. Roldan et al. (2015) showed that lamb loins meat prepared by SV method was characterized by less: brightness of color, chewiness, juiciness, toughness, and intensity flavor, as compared to roasted samples. Soletska and Krasota (2015) used boiling in water bath (at 95°C, 30 min) and SV (63°C and 65°C, 60 min) technology for chicken breast muscles. Sensory evaluation shows that SV samples had more impressive taste and smell, firm and juicy texture than those cooked in a water bath, and the color and appearance o cut resembled ham product. According to Modzelewska-Kapituła et al. (2019), the sensory quality of SV beef meat was scored higher than those subjected to steam cooking and significant differences were noted for all attributes (aroma, juiciness, tenderness, taste, overall liking). Park et al. (2020) reported that cooked chicken breast subjected to SV 60°C and SV 70°C treatments showed higher scores in overall tenderness, juiciness, and overall acceptability but lower flavor intensity than cooked meat from the control group (oven convection at 180°C). Przybylski et al. (2021) observed that in the sensory quality profile of chicken meat subjected to water bath and SV cooking are significant differences. The SV meat was more tender and juicier, had higher color tone and its overall sensory quality was rated higher by the sensory evaluation team. At the same time, this meat was distinguished by a less intense odor and flavor of cooked meat. Choi et al. (2016) used water bath cooking, oven cooking, grilling, microwave cooking, and superheated steaming for chicken breast meat. There were no significant differences in color in a water bath, oven, microwave cooked, and superheated steamed samples. The grilled samples had the highest color scores compared to the other samples. No difference in flavor scores was observed among all methods of heat treatment. Tenderness, juiciness, and overall acceptability scores were highest for the superheated steam samples, next to microwaved ones. The sensory evaluation corresponded to instrumental results of the texture of these samples. Results published by Nikmaram and Emam-Djomeh (2011) for veal muscle cooked by microwave, roasting, and braising showed that braised meat had the highest score on sensory evaluation of color and flavor. In the case of texture and juiciness, the braised and roasted samples were more tender and juicy than microwaved ones. Abdel-Naeem et al. (2021) reported that appearance, color, and flavor scores were significantly lower in boiling and microwave cooked rabbit meat compared with oven roasting, pan frying, and grilling. Moreover, tenderness and juiciness scores were significantly lower in samples cooked by boiling and pan-frying compared to grilling, microwave cooking, or oven roasting. The rabbit meat cooked by oven roasting and grilling received the significantly highest overall acceptability score, followed by the samples cooked by pan frying and microwaving, while the lowest overall acceptability score was observed in boiled ones. In Apata's (2012) experiment, the color, flavor, and overall acceptability scores were higher in fried rabbit meat than in broiled, roasted, and stewed samples. While stewed, rabbit meat was more tender and juicy than roasted, broiled, and fried. Also, Savkovic et al. (2006) used roasting and stewing for chicken meat. Stewed samples received a lower score and were tougher than roasted ones. The panelists did not state the differences in juiciness, flavor, and aroma between heat treatments. The meat was evaluated as moderately juicy, and of excellent flavor and aroma. In turn, Gök et al. (2019) noticed that roasted beef meat is characterized by a higher sensory score of taste, juiciness, and acceptability compared to sous-vide*.*

Considering all tested sensory features in current research, the overall palatability of SV goose samples was rated as excellent, S as very good and M as good.

Taking account all results it was relevant that the different heating treatment (SV, M, S) led to different physical and sensory properties of cooked goose breast muscles (with and without skin and subcutaneous fat). When considering the cooking loss, texture, color parameters, and sensory evaluation, the best method of heat treatment of goose breast muscles seems to be sous-vide cooking. In the literature on the subject, there are no studies on the heat treatment of goose meat using the sous-vide method. Therefore, in the next stage of research, there is a need to study changes in the physical properties of goose meat subjected to sous-vide technology using different combinations of temperatures and times (of thermal process), taking into account the benefits for the consumer and the producer. Thanks to the sous-vide technology, we get a product that is ready to eat, very convenient for private consumers, restaurants, and catering but this process requires a lot of energy. So, in the case of goose meat, optimal time and temperature conditions should be selected for this process.

## DISCLOSURES

I declare no conflicts of interest in publication “Comparative study on the effects of sous-vide, microwave cooking and stewing on functional properties and sensory quality of goose meat.”
